# Screening of Acrylamide Content in Commercial Plant-Based Protein Ingredients from Different Technologies

**DOI:** 10.3390/foods12061331

**Published:** 2023-03-21

**Authors:** Giacomo Squeo, Davide De Angelis, Antonio Francesco Caputi, Antonella Pasqualone, Carmine Summo, Francesco Caponio

**Affiliations:** Department of Soil, Plant and Food Science (DISSPA), University of Bari Aldo Moro, Via Amendola, 165/a, 70126 Bari, Italy; davide.deangelis@uniba.it (D.D.A.); antonio.caputi1@uniba.it (A.F.C.); antonella.pasqualone@uniba.it (A.P.); carmine.summo@uniba.it (C.S.); francesco.caponio@uniba.it (F.C.)

**Keywords:** process contamination, safety, Maillard reaction, dry fractionation, wet extraction, protein isolation, legumes

## Abstract

The demand of plant-based protein ingredients (PBPIs) in the food sector has strongly increased over recent years. These ingredients are produced under a wide range of technological processes that impact their final characteristics. This work aimed to evaluate acrylamide contamination in a range of PBPIs produced with different technologies and classified into four categories i.e., flours, dry-fractionated proteins, wet-extracted proteins, and texturized vegetable proteins. The results highlighted a remarkable variability in the acrylamide contamination in all the classes under investigation, with the flours showing the lowest mean acrylamide content (280 µg kg^−1^) compared with the wet-extracted proteins that showed the highest (451 µg kg^−1^). These differences could likely be associated with the different processing technologies used to obtain the protein ingredients. These findings suggest the need to monitor acrylamide formation during the processing of PBPIs and, consequently, to study mitigation strategies when necessary.

## 1. Introduction

Acrylamide (AA; CAS No 79-06-01) is a low-molecular-weight, α, β-unsaturated, water-soluble odorless white crystalline solid [1]. AA exists in monomeric and polymeric forms. The latter (polyacrylamide) is used primarily in industry to produce paper, paint, flocculants for drinking water clarification, and to produce electrophoresis gels [2]. The monomeric form has been classified since 1994 as a probable human carcinogen (Group 2A) by the International Agency for Research on Cancer [2] and, in April 2002, researchers from the Swedish National Food Administration and the University of Sweden reported for the first time the presence of monomeric acrylamide in starchy foods cooked at high temperatures [3].

Since then, numerous studies were carried out to investigate both the toxic effects and the mechanisms of acrylamide formation in foods. Medical studies have reported that after ingestion, acrylamide enters the bloodstream and reaches various tissues in the body affecting their metabolism, reproduction, and normal cell division [4].

Considering acrylamide formation in foods, early studies have shown that it is primarily related to the Maillard reaction [5], starting from the interaction between the amino groups of free amino acids (especially asparagine) and the carbonyl groups of reducing sugars (glucose and fructose). The Maillard reaction is triggered by high temperatures and low moisture content [6]. It was reported that asparagine is the main precursor of AA. Moreover, glutamine and methionine lead to the production of AA, but in smaller amounts [7]. Other possible pathways include the thermal degradation of acrolein or aspartic acid, carnosine, and β-alanine [5,8,9,10], and the pyrolytic acrylamide formation in wheat gluten [11]. A global meta-analysis highlighted a high variability in the acrylamide content in food products, showing a higher content in potato-based food (740.33 μg kg^−1^), followed by fried foods, breakfast cereals, and coffee, while nuts presented the lowest amount [12].

Recently, in the European Union, the content of acrylamide in French fries, sliced potato crisps from fresh potatoes, snacks, crackers, and other potato products from potato dough, bread, breakfast cereals, fine bakery wares, coffee and coffee substitutes, and baby food, has been regulated [13]. Numerous reports about acrylamide content in these common food products could be found in the literature [12,14,15]; however, there is a plethora of innovative food products and ingredients of which no information is available. Among them, plant-based protein ingredients (PBPIs) are worthy of attention due to their impressive growing rate in the market worldwide. In fact, investments in the alternative protein sectors are constantly increasing [16]. However, owing to their fast introduction in the market, there is a lack of evidence of the nutritional value and data on the health impact of these products [17]. Nowadays, PBPIs include: (i) native legume flours (e.g., soy, pea, chickpea, and fava bean flours); (ii) protein isolates and concentrates derived from cereals (e.g., wheat or oat), legumes, other pseudo cereals, and seeds (e.g., quinoa, hemp, and sunflower) [18,19]. Protein isolates and concentrates are obtained by different extraction technologies such as wet extraction, which consists of a series of extractions by chemicals and water with a final drying stage [20]; or the dry fractionation technology, a solely physical concentration without any direct heating process and any use of water and chemicals [21]. Moreover, texturized vegetable proteins (TVPs) are commonly obtained by the extrusion cooking of protein raw materials [22,23]. PBPIs and related products are rapidly introduced in the market, leading to possible concerns related to the capacities of policymakers and regulatory bodies to keep up with such expeditious development [17].

To the best of the authors’ knowledge, the content of acrylamide in PBPIs and their derived products has not been investigated yet. Moreover, only few reports are available considering legume-based products [24,25]. The various technological processing methods used in PBPIs production [20,23,26,27] may cause stresses to the raw materials, influencing the formation of AA. In particular, we hypothesized that, in the context of PBPIs production, AA could be developed: (i) during the protein concentration/isolation, owing to the application of high temperatures in the drying stage; (ii) during the extrusion-cooking process in which high temperatures are required to obtain the texturization of the protein; and (iii) during other drying and milling stages applied to the raw materials.

Therefore, the aim of this research is to provide a preliminary report on the AA content in PBPIs available in the market, considering some specimens of native legume flours, PBPIs obtained by wet extraction and dry-fractionation, and TVPs produced by extrusion-cooking.

## 2. Materials and Methods

### 2.1. Chemicals and Reagents

*D_3_*-acrylamide, acrylamide, anhydrous magnesium sulfate (MgSO_4_), hexane, methanol, formic acid, and acetonitrile LC-MS grade were purchased from Merck Life Science S.r.l. (Milano, Italy). Sodium chloride (NaCl) was purchased from ITW Reagents, S.r.l. (Monza, Italy). A 2 mL DisQue^TM^ extraction tube containing 50 mg of PSA and 150 mg of MgSO_4_ were purchased from Waters S.p.a. (Sesto San Giovanni, Italy). A certified reference material (Acrylamide in crispbread, sample no. 390, ERM^®^-BD272) was purchased from BAM (Berlin, Germany). Ultrapure water was produced by an Elga Purelab Option R system (Veolia Environnement S.A., Paris, France).

### 2.2. Plant-Based Protein Ingredients Collection

The list of the materials used for this study is reported in Section 3. A total of 17 PBPIs of different origin and type were collected and then analyzed for acrylamide content. The specimens were classified according to the information provided by the suppliers into 4 categories as follows: 3 native legume flours (F), including 2 types of red lentil (*Lens culinaris* Medik.) and green mung bean (*Vigna radiata* (L.) R. Wilczek); 6 dry-fractionated protein concentrates (DF), i.e., beige chickpea (*Cicer arietinum* L.), green pea (*Pisum sativum* L.), red lentil, fava bean (*Vicia faba* L.), and green mung bean; 6 protein concentrates/isolates produced by wet extraction (WE), i.e., 2 chickpea proteins, 2 soy proteins (*Glycine max* (L.) Merr.), wheat gluten (*Triticum aestivum* L.), and oat (*Avena sativa* L.); and 2 texturized vegetable proteins granulates (TVPs), produced by extrusion-cooking of yellow pea protein and sunflower protein (*Helianthus annuus* L.). F, DF, and WE were in the form of powdery samples, whereas the TVPs were granulate pellets. The samples (one lot per each) were purchased by different suppliers selling in European countries. The moisture content of the samples was within the range of 5–9% (data not shown). All the samples, once collected, were directly stored in the original state at −20 °C until the analysis.

### 2.3. Acrylamide Extraction

Acrylamide extraction was carried out according to Mastovska and Lehotay [28] with minor modifications. The flours and the powdery concentrated proteins (DF and WE) were treated as such, while the textured proteins (TVPs) were finely ground and sieved through a 0.6 mm sieve.

About 1 g of the sample was weighted into a 50 mL centrifuge tube; then, *d_3_*-acrylamide (100 μL of 2.5 µg mL^−1^ standard solution) and 5 mL of hexane were added and the tube vortexed. Afterwards, 10 mL of deionized water and 10 mL of acetonitrile were added followed by the addition of 4 g anhydrous MgSO_4_ and 0.5 g of NaCl prepared before each analysis in a laboratory shuttle. The tube was immediately closed and vigorously shaken for 1 min with an agitator (Multi Reax, Heidolph Instruments GmbH & Co., Schwabach, Germany). The tube was then centrifugated for 5 min at 3500 RCF (SL 16R Centrifuge, Thermo Fisher Scientific, Waltham, MA, USA) and the hexane layer discarded. An amount of 1 mL of the acetonitrile extract was transferred to the d-SPE tube, shaken for 30 s, and then centrifugated at 7580 RCF (Heraeus Biofuge Pico, Newport Pagnell, U.K.) for 1 min. The supernatant was finally collected by using a syringe, filtrated with 0.22 µm nylon filters (Sartorius Stedim Biotech Gmbh, Göttingen, Germany), and diluted with the LC mobile phase (1:1, *v*/*v*) directly into the vial for LC-MS analysis. Extractions were performed in triplicate for each sample (*n* = 3).

### 2.4. LC-MS Analysis

AA analysis was performed using a Dionex Ultimate 3000 RS UHPLC system (Thermo Fisher Scientific, Waltham, MA, USA) consisting of an LPG-3400RS quaternary pump, WPS-3000 TRS autosampler, TCC-3000RS column compartment maintained at 30 °C, interfaced with an electrospray ionization chamber (H-ESI), and an LTQ Velos Pro linear ion trap mass spectrometer (Thermo Fisher Scientific, Waltham, MA, USA). The sample injection volume was 5 µL and the stationary phase was a Hypersil Gold aQ C18 column (100 × 2.1 mm, particle size 1.9 μm). An isocratic elution was carried out by a mobile phase of H_2_O-methanol (99.5:0.5, *v*/*v*) at a constant flow rate of 200 µL min^−1^ for 4 min followed by a post-analysis washing step (at 200 µL min^−1^ for 4 min) using 0.1% formic acid in acetonitrile–methanol (50:50, *v*/*v*) and a 5 min equilibration time at the initial conditions. The retention time of AA was 1.6 min.

Data were acquired by positive ionization mode (ESI+) using the optimized instrument parameters obtained from the tuning procedure. The MS conditions were capillary temperature 320 °C; source heater temperature 250 °C; nebulizer gas N_2_; sheath gas flow 35 psi; auxiliary gas flow, 13 arbitrary units; and capillary voltage 3.5 kV, S-Lens RF Level 33%. The transitions *m/z* 72 → 55 and 75 → 58 were used for acrylamide and for *d_3_*-acrylamide quantitation, respectively. The CID energy was 32, the activation time and activation Q were 10 ms and 0.500, respectively. MS data were acquired and processed using Xcalibur v.2 (Thermo Fischer Scientific, Waltham, MA, USA). A signal–ratio calibration curve was built up using the relative response of acrylamide vs. *d_3_*-acrylamide (i.e., the ratio between the peak area of the acrylamide external standard and the area of *d_3_* internal standard). The calibration range was 1–230 ng mL^−1^ (R^2^ = 0.999) (Figure S1) and the limit of detection (LOD) and limit of quantitation (LOQ) were 7 ng mL^−1^ and 24 ng mL^−1^, respectively. The accuracy of the method was verified by a certified reference material (CRM). To account for instrumental random errors, each AA extract was injected in duplicate.

### 2.5. Statistical Analysis

The results of the AA content have been expressed as µg kg^−1^ on fresh weight basis and reported as mean ± standard deviation from three independent replicates (*n* = 3). Then, the data were subjected to one-way analysis of variance (ANOVA). Previous to this stage, the normality of the data was checked by the Ryan–Joiner test which showed that the data did not follow a normal distribution. Thus, the Johnson transformation was applied to effectively transform those into normally distributed data. Afterward, ANOVA was carried out followed by the Bonferroni test for multiple comparisons at a significance level α = 0.05. The descriptive statistics per each PBPI class were calculated using the mean value per each sample of the class. The statistical elaboration was carried out in Minitab 17 (Minitab Inc., State College, PA, USA).

## 3. Results

The mean content of AA determined in the 17 samples is reported in Table 1, while in Table 2 the descriptive statistics of the dataset are reported. The specimens considered in this study cover the most important protein sources used in the food industry as ingredients for plant-based food products [29], including both cereal-based proteins (i.e., wheat and oat) and legume-based proteins (i.e., pea, chickpea, fava bean, and lentil). The protein content (stated on the labels) ranged from 25 g 100 g^−1^ to above 90 g 100 g^−1^ on a fresh weight basis.

**Figure 1:**
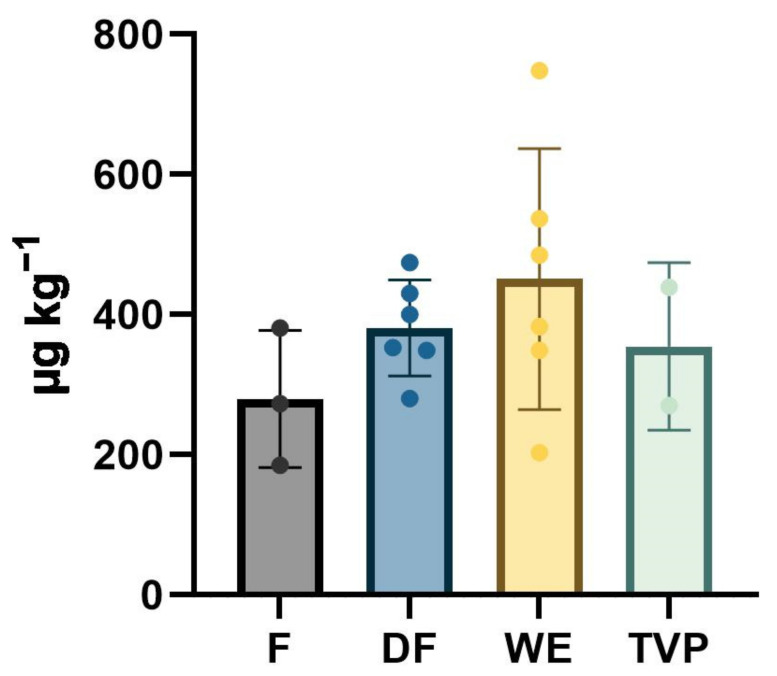
Bar chart of the acrylamide level in the investigated PBPIs divided by classes. F, flours; DF, dry fractionation; WE, wet extraction; TVP, texturized vegetable protein.

The AA content was highly variable in the PBPIs under investigation, even when considering the same species. Specifically, the highest significant AA content was found in one of the chickpea WE ingredients. In descending order, it was followed by two other WE samples, namely, the PBPIs obtained from wheat gluten and from oat. Then, lower contents were progressively found for the other ingredients, without any clear pattern linked to the processing technology, the protein source, nor the protein content. In fact, a great variability was found between each class, as can be observed in Table 2 and Figure 1.

Overall, the F class showed the lowest mean value of AA content (Table 2, Figure 1). In this class, the mung bean F showed the significantly lowest AA concentration of the whole dataset (Table 1). The AA content of native flours has been scarcely assessed. Shih et al. [30] reported a content ranging from 98 µg kg^−1^ to 115 µg kg^−1^ in cereal flours from rice, corn, and wheat, whereas Žilić et al. [31] reported that the acrylamide content in different wheat, rye, and maize raw flours were below the LOQ. To the best of the authors’ knowledge, no references were found regarding the AA content of native legume flours, making a comparison with our results difficult. However, the legume flours analyzed in this work showed roughly the same magnitude in AA with respect to the report of Shih et al. [30], although in the lentil samples the content was about two to three times higher.

Therefore, the origin of the AA formation in native legume flours should be better investigated. To date, we can only suppose that post-harvest treatments such as drying, as well as the milling operations, could cause an increase in temperature which leads to the development of AA. In this regard, Taeymans and colleagues [32] have showed that even temperatures ≤ 80 °C during biscuit baking can promote the formation of AA.

The concentrated proteins considered in this study (i.e., DF and WE) showed a similar minimum value of AA content, whereas the maximum and, consequently, the range and the mean content was much higher for the WE class (Table 2, Figure 1).

This trend could be explained by the differences in the WE processing technologies used to obtain the proteins, which are more complex and variable [33] compared with the dry fractionation [21]. Indeed, the wet extraction process consists of several unit operations, and it is possible that the different drying technologies and the temperatures used after the protein extraction [26] can have a major influence on the acrylamide formation. As a proof of that, it is interesting to note (Table 1) that comparing two different WE proteins obtained by the same species (chickpea) and having the same protein content (>90%), one had roughly twice as much AA (748 µg kg^−1^) as the other (383 µg kg^−1^).

At the same time, the raw materials themselves, which conceivably contain different levels of precursors, could influence AA development [31,34]. For example, a soy protein WE showed the lowest content of AA, similar to the mung bean F (Table 1). This result indicates that the type of raw material, as well as the production technologies, have an influence on the AA development in these products. In turn, different levels of precursors in raw materials could be related to varietal factors, growth areas, and agronomic practices [35]. Recently, Hasan et al. [36] found a large variability in nine different classes of foods and this variability was tentatively associated with the different pathways that may lead to AA development, as well as to the different levels of precursors. According to our observations, the authors also stated that the different sources of the ingredients and divergent food processing systems might have a remarkable effect in AA formation [36]. Further investigation would be useful to better highlight the relationships between agronomic, compositional, and processing variables in the formation of AA in PBPIs.

Finally, similar conclusions could be drawn for the TVP class (although in this study only two samples were considered), whose AA content was very variable and significantly higher for the pea protein compared with the sunflower one (Table 1). Again, this result could be explained by the multitude of extrusion processing conditions that can be used to produce TVPs [37]. Indeed, as reported in previous studies, the processing conditions during extrusion cooking of PBPIs can widely vary in terms of temperature (which is always above 100 °C and can reach 180 °C), residence time (up to 3 min), and moisture content (20–35% in low-moisture extrusion and 40–70% in high-moisture extrusion) [23,27], thus influencing the AA formation.

These results present a preliminary screenshot of AA contamination in PBPIs; however, considering the great variability found, generalizing these observations could be misleading. A comprehensive understanding of the problem, followed by the definition of proper mitigation strategies (if needed), will support the implementation of new studies aimed at unravelling the impact of agronomical practices, raw material composition (especially for legumes), and processing variables (temperature, time, moisture, etc.) on AA formation during PBPIs production.

## 4. Conclusions

Acrylamide is a dangerous process contaminant whose content should be carefully monitored and, if present, reduced as much as possible. In this study, some plant-based protein ingredients were investigated and the results showed that they suffer from acrylamide contamination. The contamination is widely variable between native legume flours, concentrated proteins, and texturized proteins. This evidence suggests that these ingredients and related plant-based foods deserve attention. Critical stages during processing should be identified, and then proper mitigations strategies should be applied.

Finally, considering the increasing demand and utilization of PBPIs, it is necessary that policymakers and regulatory authorities consider monitoring and regulating such ingredients.

## Figures and Tables

**Table 1:** List of the plant-based protein ingredients (PBPIs) with their relative acrylamide content and the results of the statistical analysis.

Type	Protein Source	Protein Content on the Label (g 100 g^−1^)	ACRYLAMIDE (µg kg^−1^)	
F	Lentil (Supplier 1)	29	381 ± 49	de
F	Lentil (Supplier 1)	28	273 ± 14	fg
F	Mung Bean (Supplier 1)	25	185 ± 7	g
DF	Chickpea (Supplier 2)	50	474 ± 44	bcd
DF	Pea (Supplier 2)	55	430 ± 36	cde
DF	Lentil (Supplier 2)	55	400 ± 22	cde
DF	Fava bean (Supplier 2)	55	353 ± 26	ef
DF	Mung Bean (Supplier 2)	56	349 ± 20	ef
DF	Lentil (Supplier 2)	65	280 ± 30	fg
WE	Chickpea (Supplier 3)	>90	748 ± 42	a
WE	Wheat gluten (Supplier 4)	80	537 ± 52	b
WE	Oat (Supplier 5)	55	485 ± 21	bc
WE	Chickpea (Supplier 3)	>90	383 ± 29	de
WE	Soy (Supplier 6)	60	349 ± 26	ef
WE	Soy (Supplier 7)	87	203 ± 10	g
TVP	Pea (Supplier 6)	80	439 ± 48	cd
TVP	Sunflower (Supplier 8)	80	270 ± 15	fg

The results are presented as mean ± standard deviation (*n* = 3). Different letters between the samples indicate significant differences in the acrylamide content at α = 0.05. F, flours; DF, dry fractionation; WE, wet extraction; TVP, texturized vegetable protein.

**Table 2:** Descriptive statistics of the acrylamide content in plant-based protein ingredients (PBPIs) grouped per class.

PBPI Class	N	Mean	SD	RSD	Min	Max	Range	Median	Q1	Q3	IQR
F	3	280	98	35	185	381	196	273	229	327	98
DF	6	381	69	18	280	474	194	376	350	423	73
WE	6	451	186	41	203	748	545	434	357	524	167
TVP	2	354	119	34	270	439	169	354	312	396	84

F, flours; DF, dry fractionation; WE, wet extraction; TVP, texturized vegetable protein. N, number of independent samples per each class; SD, standard deviation; RSD, relative standard deviation; Q1, first quartile; Q3, third quartile; IQR, interquartile range. All the results are expressed in µg kg^−1^ except RSD, which is expressed as %. The descriptive statistics were calculated starting from the AA mean value observed per each sample, as reported in Table 1.

## Data Availability

Data are contained within the article.

## Supplementary Materials

The following supporting information can be downloaded at: https://www.mdpi.com/article/10.3390/foods12061331/s1, Figure S1: LC-MS calibration curve developed for the quantitation of acrylamide. Each standard solution has been analysed in duplicate.
