# Supplementary material for: Screening of Acrylamide Content in Commercial Plant-Based Protein Ingredients from Different Technologies
Source: Foods. 2023 Mar 21;12(6):1331. doi: 10.3390/foods12061331 (PMC10048331; doi:10.3390/foods12061331)
Supplement: Supplementary file 1 [file foods-12-01331-s001.zip › foods-2259352-supplementary.pdf]

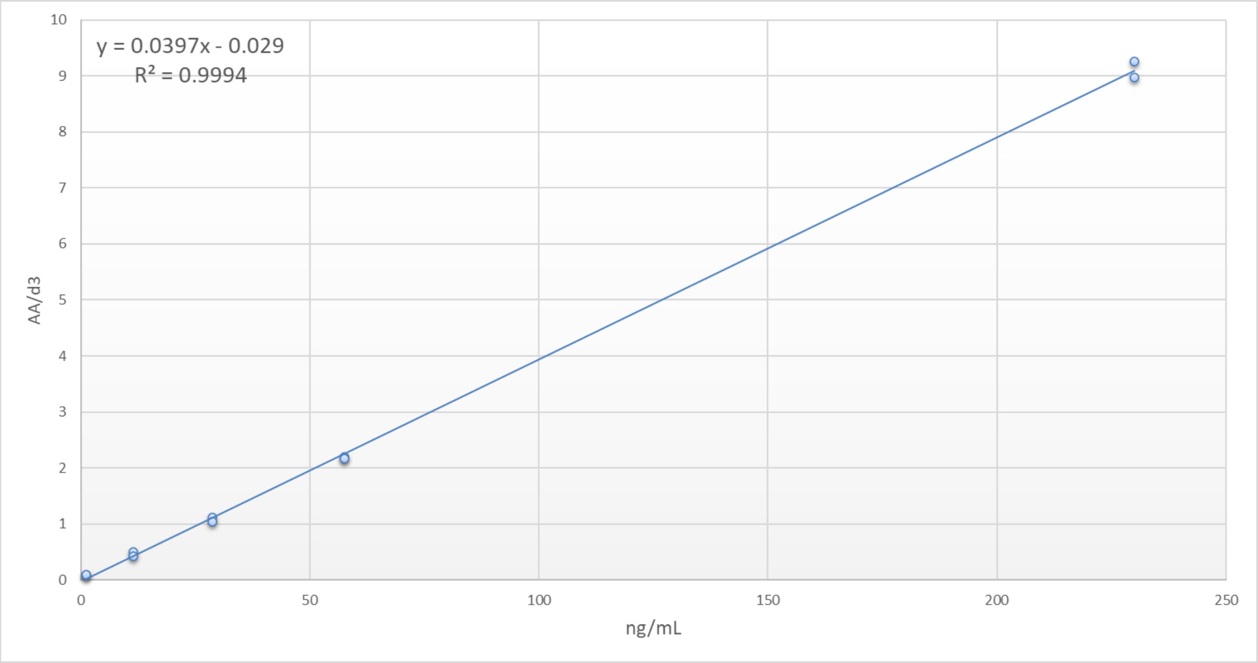

Figure S1. LC-MS calibration curve developed for the quantitation of acrylamide. Each standard solution has been analysed in duplicate.
